# Supplementary material for: Endurance Exercise Training Mitigates Diastolic Dysfunction in Diabetic Mice Independent of Phosphorylation of Ulk1 at S555
Source: Int J Mol Sci. 2024 Jan 3;25(1):633. doi: 10.3390/ijms25010633 (PMC10779281; doi:10.3390/ijms25010633)
Supplement: Supplementary file 1 [file ijms-25-00633-s001.zip › ijms-2734595-supplementary.pdf]

Endurance Exercise Training Mitigates Diastolic Dysfunction in Diabetic Mice Independent of Phosphorylation of Ulk1 at S555

Yuntian Guan [1,2,3], Mei Zhang [1,2,4], Christie Lacy [1], Soham Shah [5], Frederick H. Epstein [5] and Zhen Yan [1,2,3,4,5,6,7,*]

[1]Fralin Biomedical Research Institute, Center for Exercise Medicine Research at Virginia Tech Carilion, Roanoke, VA 24016, USA; yg6ju@virginia.edu (Y.G.)
[2]Center for Skeletal Muscle Research at Robert M. Berne Cardiovascular Research Center, University of Virginia, Charlottesville, VA 22903, USA
[3]Departments of Pharmacology, School of Medicine, University of Virginia, Charlottesville, VA 22903, USA
[4]Departments of Medicine, School of Medicine, University of Virginia, Charlottesville, VA 22903, USA
[5]Departments of Biomedical Engineering, School of Medicine, University of Virginia, Charlottesville, VA 22903, USA; ss8ma@virginia.edu (S.S.); fhe6b@virginia.edu (F.H.E.)
[6]Departments of Molecular Physiology and Biological Physics, School of Medicine, University of Virginia,
Charlottesville, VA 22903, USA
[7]Department of Human Nutrition, Foods, and Exercise, College of Agriculture and Life Sciences, Virginia Tech, Blacksburg, VA 24061, USA

This file contains the supplementary Figure S1-3.

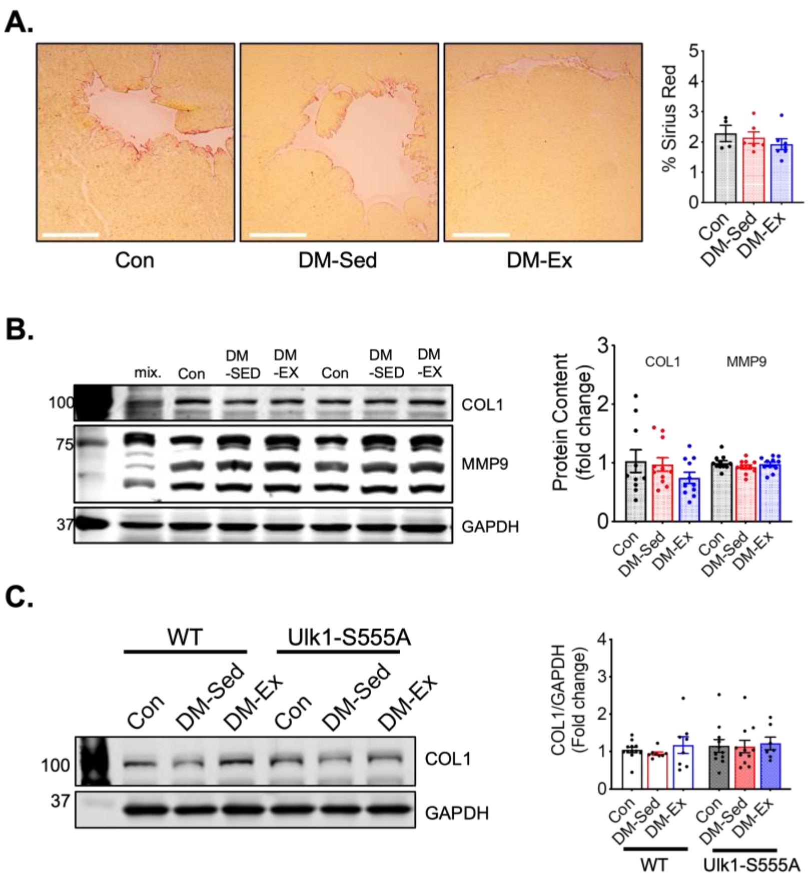

Fig S1. HFD-STZ-diabetes does not induce significant cardiac fibrosis. (**A**)**:** Representative images and quantifications of picro-sirius red staining. n = 4, 6, 6 for Con, DM-Sed and DM-Ex groups, respectively. scale bar = 500 µm. (**B**)**:** Western blot assessment of Col1 and Mmp9 in wildtype whole-heart lysates. n = 10 – 12. (**C**)**:** Western blot assessment of Col1 in WT and *Ulk1-S555A* mice. n = 8 – 12.

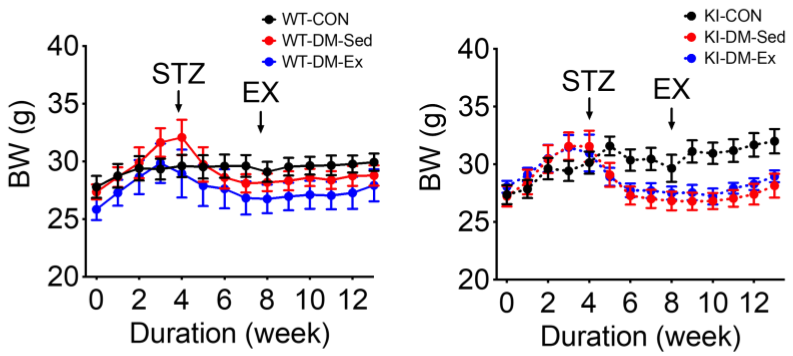

Fig S2. Body weight of WT and *Ulk1-S555A* mice. STZ, beginning of injections of STZ; EX, beginning of the 6 weeks of voluntary wheel intervention.

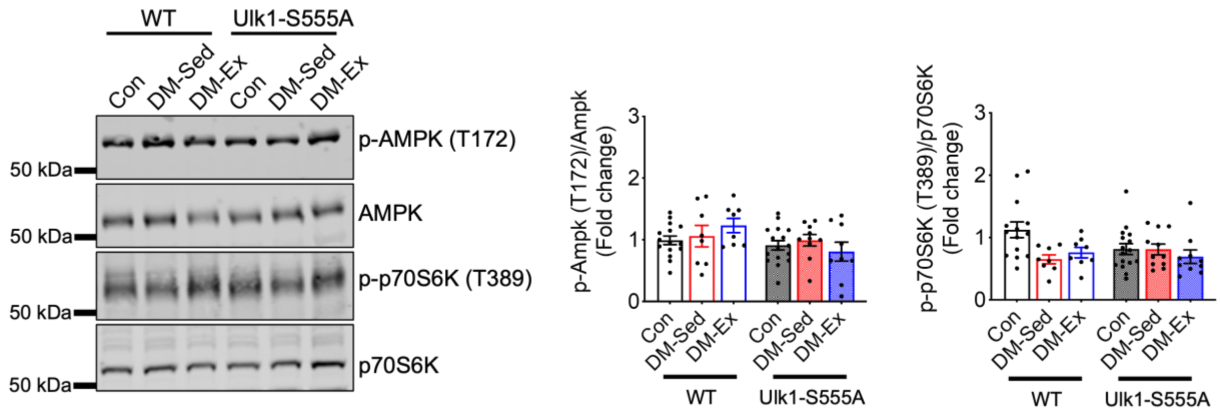

Fig. S3. AMPK and mTOR signaling in the Ulk1-S555A diabetic mice. Western blot assessment of phosphorylated AMPK (T172) and p70S6K (T389) in WT and *Ulk1-S555A* mice. No significant differences were detected. n = 10 – 16.
